# Educational Programs for the Promotion of Health at School: A Systematic Review

**DOI:** 10.3390/ijerph182010818

**Published:** 2021-10-14

**Authors:** David Pérez-Jorge, María Alejandra González-Luis, María del Carmen Rodríguez-Jiménez, Eva Ariño-Mateo

**Affiliations:** 1 Department of Didactics and Educational Research, Faculty of Education, University of La Laguna, San Cristóbal de La Laguna, 38200 Santa Cruz de Tenerife, Spain; alejandragonzalezluis9@gmail.com (M.A.G.-L.); mcrojime@ull.edu.es (M.d.C.R.-J.); 2 Department of Psychology, European University of Valencia, 46010 Valencia, Spain; EVA.ARINO@universidadeuropea.es

**Keywords:** health, health promotion program, primary school, secondary school, compulsory education

## Abstract

Context: Health promotion programs generate healthy changes in the educational community. However, not all of them meet the expected objectives due to multiple factors that affect their development, such as the teachers overload work, the lack of specific training, the lack of time to carry out health promotion activities, the lack of flexibility of the programs, and their non-inclusion in the training programs of the centers. Objective: To know the scope of the strategies and programs that promote healthy habits among students in compulsory educational stages. Data sources: a systematic review of articles in English, using the Web of Science (WOS), Medline, and PsycINFO databases.

## 1. Introduction

Health Promoting Schools (HpS) are resources for training, which are currently becoming a fundamental element for improving the comprehensive education of students, especially in the field of health. These schools favor the acquisition of knowledge and skills so that students are able to reflect and make decisions, in order to improve their health. These centers acquire a commitment to the development of training actions and to the implementation of programs for the promotion of healthy habits, creating a related awareness shared by all members of the educational community.

### 1.1. The Concept of Health

This work involves making a prior reflection on what is understood by health within the framework of the competencies that must be promoted from a school for its achievement. We start from the concept most agreed on and shared by the educational community, taking as a reference the definition from [1], which understands that health is the state of physical, mental, and social well-being of each individual and that it is a fundamental right of all citizens, without distinction of race, religion, political ideology, or economic or social conditions.

There are several approaches that have guided intervention in the field of health, as highlighted by [2]: (a) the preventive model, focused on early intervention; (b) the sanitary model, centered on the control of biophysical environmental conditions and; (c) the socio-medical model, focused on intervention in social and relational contexts. From our point of view, it is important to focus on the latter, since, as [3], this model relates the social, economic, and political context with the lifestyles of individuals. In this sense, it is considered essential to focus actions on health promotion, disease prevention, healing, and rehabilitation of people.

In this sense, [4] confirms that health forms a style and philosophy of life typical of each person and that it implies the promotion of autonomy that favors the configuration of one’s own personality. In this way, the individual is able to make their own decisions through a process of responsible reflection that helps them to adopt the habits and customs in solidarity with other citizens and with themselves, which has a positive impact on the enjoyment and improvement of their quality of life.

### 1.2. Health Promotion and Health Promoting Schools

In 1978, WHO, UNESCO, and UNICEF proposed various initiatives to introduce health promotion in schools. Years later, in 1986, the Ottawa Charter was drawn up, which recognized the importance of health education in achieving the wellbeing of all individuals [5]. Gradually, the promotion of health and healthy lifestyles has been promoted, in order to prevent diseases and improve people’s quality of life, which is why schools have taken a leading role in the promotion of health [6]. Reference [7] defines health promotion strategies in the school environment as a set of activities focused on improving the health of the entire educational community, and alludes to the need to influence the physical and social environments and policies of health promotion through the use of appropriate methodologies and school programs that promote their development. It is essential that the entire educational community be part of the health promotion process [8], therefore the need for health literacy arises, with the intention that people acquire knowledge and skills that allow them to promote health appropriately [9].

From the importance attributed to centers in the promotion of health, Health Promoting Schools (HpS) emerged, which oversee achieving social, economic, and environmental changes in the population in which they intervene. The essential function of HE is to develop the capacity of the entire educational community to achieve healthier lifestyles. These schools are in charge of carrying out activities that improve the health of the entire educational community, providing them with knowledge and habits for comprehensive care of people’s health. Reference [7] argues that the improvement of health states leads to an improvement in the academic results of the students.

Authors of [10] believe that it is essential to create policies that promote healthy lifestyles and prevent diseases, which should be promoted from educational centers as considered by the Ministry of Education, Culture, and Sports of the Government of Spain. Educational centers have direct access to students and the entire educational community, and therefore can directly influence the development of healthy habits and lifestyles. Educational centers are essential for the comprehensive development of students and the promotion of healthy lifestyles both physically, mentally, and socially.

As [7] underlines, HE aims to improve both the health of their students and their academic level through teaching and learning experiences and focused on actions related to well-being and healthy lifestyles. These actions are focused on elements that can affect health states, specifically referring to healthy eating, physical activity, emotional wellbeing, the consumption of drugs, tobacco, or alcohol, as well as the irresponsible use of Technology of Information and Communication (ICT), and the environment of the center.

### 1.3. Programs That Promote Health

An educational program is a set of activities that provides knowledge, skills, abilities, and competencies to students. Programs that promote health also have the objective of developing health in the educational community [11]. It is a teaching–learning process in which health is worked on and the quality of life of people is promoted, allowing critical thinking, affectivity, problem-solving, and social relationships develop [12]. According to [13], it is common for health risk behaviors to be seen in students, therefore, their prevention is essential through school programs that train and educate students. Reference [14] adds that educational centers are responsible for promoting health through programs, since through them healthy behaviors and habits are learned, thus avoiding risky behaviors. They state that health must be worked on throughout life, from childhood to adulthood, if a healthy culture and lifestyle is to be consolidated. The early approach and the consolidation of habits due to the influence effect are two of the reasons why educational centers are considered the most appropriate spaces to promote health [12].

The contribution of [15] affirms that for a health program to work, it is necessary that it be adapted to the context in which it is going to be implemented, moreover, they consider that this aspect is key if educational centers are to be a true HpS. The programs should not be implemented as something specific that is developed in certain circumstances, since health promotion must be part of the ideology and culture of the center, it must be a priority that must be addressed daily in the classroom, either as a specific subject [16] or in a transversal way [17]. Its value and relevance should prioritize its approach, giving time and opportunities for its internalization [16]. An example of the limited effects of specific actions in the field of health education is the study of [18], whose changes, despite being significant, were not maintained over time. The changes were observed with regard to healthy behaviors and knowledge about health, however, there were no changes in eating habits, nor in physical activity.

The main objective of this study is to know the scope of the strategies and programs that promote healthy habits among students in compulsory educational stages, using a systematic review methodology. We are unaware of the existence of previous systematic reviews, and this is a topic of great importance and relevance. For this reason, we consider it useful to identify the effectiveness of health promotion in HE.

Analyzing the existing theoretical framework, the research questions are:

- What programs do health-promoting schools develop and promote?
- Are educational programs effective in promoting healthy habits in students?
- What are the difficulties and limitations for the development of programs that promote health in educational centers?

## 2. Materials and Methods

The programs that promote health in educational centers tend to focus on different areas, therefore, studies that address specific areas of health promotion and not only those that speak on health promotion in a generic way were accepted. In addition, all those who are part of the educational community (students, teachers, principals, families) were considered as participants. Studies on health promotion programs had to provide data on the benefits and/or limitations of the implementation of these programs, whether of a preventive or specific interventional nature. Therefore, at the beginning of this research, a series of criteria were established for the inclusion and exclusion of documents.

In this way, the criteria considered are shown in Table 1:

### 2.1. Literature Review

A systematic review of the scientific literature focusing on health promotion programs in schools was carried out for this study. This type of study aims to know, through the systematization of the search for sources and studies, the state of research in relation to a topic or question of research [19] This study was developed using the PRISMA (Preferred Reporting Items for Systematic Reviews and Meta-Analyses) declaration model for meta-analysis and systematic review studies [20]. The PRISMA statement guides the conceptual and methodological aspects considered during the development of systematic review studies [21]. It is a type of study that analyzes the scientific literature on a topic with the aim of constructing valid and objective conclusions [22]. This is not only a study that provides knowledge on how health promotion is carried out in schools, but also poses challenges that will open the way for new studies and research.

The search of the sources was carried out on the databases of the Web of Science (WOS), which compiles the most important articles in the educational field, in addition, the search was carried out on Medline and PsycINFO, as they collect studies most prominent in the field of health. The search for sources lasted approximately 4 weeks, beginning on 24 April 2021, and ending on 26 May of the same year.

The following terms were used to search the indicated databases: “Health promotion program” together with a combination of educational terms (“Early childhood education”, “Primary school”, “Secondary school”, “Compulsory education”, “obligatory education”, “primary education”, “secondary education”, “basic education”, “elementary school”, “early education” and “high school”). It should be noted that the search was limited to research articles in English.

### 2.2. Characteristics of the Included Studies

Initially, the search strategy used was too general and non-specific, obtaining too many documents that were not related to the objective of the work. This search strategy was used: (School OR “obligatory education”) AND “health promotion program”.

In order to refine and focus the search, the search terms were broken down and replaced with synonyms. On the one hand, “health promotion program” was maintained as it is the main focus of this search and, in turn, all other terms were selected and debugged. The terms “Early childhood education”, “Primary school” and “Secondary school” were alternated between. With these combinations of topics and Boolean operators, the search offered a number of suitable articles to start selection and subsequent analysis. However, it was considered necessary to add the synonyms of the primary topics. As a result, after determining the final selection and alternating different combinations with Boolean operators, the final search was established with: (“Early childhood education” OR “Primary school” OR “Secondary school” OR “Compulsory education” OR “obligatory education” OR “primary education” OR “secondary education” OR “basic education” OR “elementary school” OR “early education” OR “high school”) AND “health promotion program”.

### 2.3. Procedure

The eligibility assessment was carried out independently and standardized. To do this, we began by searching the three databases mentioned above and, using the Mendeley bibliographic manager, all documents that were duplicated were eliminated. After this, the inclusion criteria, indicated above, were applied, eliminating those documents that did not meet the requirements, for which it was necessary to read the titles and summaries of all the documents. Finally, a complete reading of the remaining documents was made to confirm that they met the objectives of the study.

To extract the necessary information from the reports, the Atlas.ti V. 7 (Qualitative analysis program, originated at the Berlin University of Technology, Berlin, Germany in a project called ATLAS, between 1989 and 1992. The acronym stands for Archiv für Technik, Lebenswelt und Alltagssprache) program was used, with which all the important information was selected and encoded to be accessed quickly and easily. The information extracted refers to the main characteristics of each health promotion program, the sample, and the country in which the program was carried out, as well as the educational level at which it was put into practice. Likewise, the study methodology (qualitative or quantitative) and the main results were extracted, in order to know its limitations and benefits.

## 3. Results

### 3.1. Study Selection

**Figure 1:**
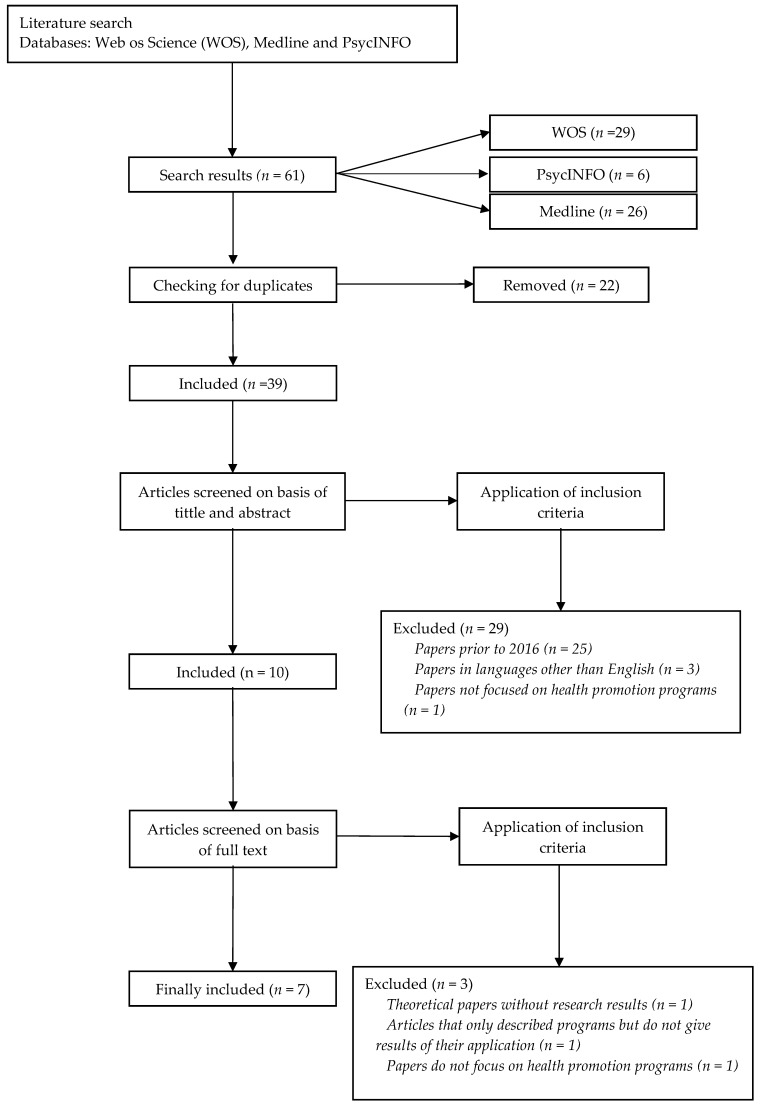
Flow chart document.

We began by searching the databases, where 29 documents were identified in WOS, 26 articles in Medline, and 6 in PsycINFO. Therefore, the search resulted in a total of 61 documents. At this point, duplicates were eliminated using the Mendeley bibliometric manager, excluding a total of 22 articles. After this, the inclusion criteria were applied, eliminating 25 documents that were not written in the last 5 years (2016–2021) and 3 articles that were not in English. The titles and abstracts of the documents were then read, eliminating one that did not specifically deal with health promotion programs. Finally, there were a total of 10 documents that were read completely, after the complete reading, 3 that were not research studies on the application of programs were discarded. Finally, as can be seen in Figure 1, the total number of documents to be analyzed was seven.

### 3.2. Characteristics of the Included Studies

The seven articles selected for the review were research studies published in English and between the years 2016 and 2021, thus ensuring an analysis of the results on the application of programs for health promotion was updated. All the studies were international, carried out mainly in European countries (Ireland, Austria, Scotland, and Germany) and also in Australia, the United States of America, and Iran. According to the methodology used to approach the study, it was confirmed that two used qualitative methodology (28.57%; *N* = 2), another two quantitative (28.57%; *N* = 2), and three were mixed studies (42.85%; *N* = 3).

**Figure 2:**
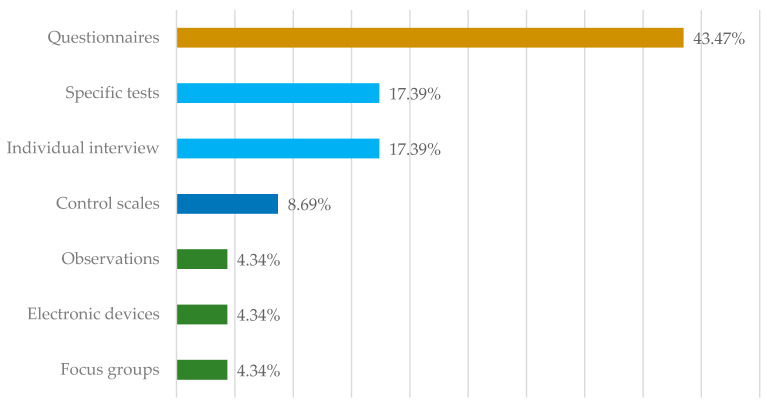
Evaluation instruments used in the different publications.

The evaluation instruments used were diverse, with more than one instrument in each of the studies, including: individual interviews (17.39%), questionnaires (43.47%), observations (4.34%), specific tests (skills and physical performance) (17.39%), focus groups (4.34%), electronic devices for routine and habit control (4.34%), and control scales (8.69%). As can be seen in Figure 2, the most widely used were questionnaires, interviews, and tests.

In most of the studies, the selected samples were students from educational centers; only one study focused solely on the views of teachers on the importance of health promotion in schools. This shows a significant weakness in the approaches of the interventions that are developed in schools. These are usually one-off actions carried out by health professionals in which teachers are hardly involved (e.g., vaccination campaigns, oral hygiene campaigns…).

Regarding the results, it should be noted that there are disparities, as some programs did meet the objectives that had been set while others did not meet expectations, and their application was not very effective. Likewise, three studies focused on the primary education stage (42.85%), three on compulsory secondary education (42.85%), and one on both stages (14.28%). As can be seen from the distribution of studies at the educational stage, these studies mainly focused on the stages of primary and secondary education. All the information can be seen in Table 2, where the author or authors, year of publication, purpose, design, sample, evaluation instrument, type of program, and main results are also listed.

### 3.3. Identification of Health Promotion Programs

Each of the selected studies worked on different types of programs to promote health in educational centers. The programs mainly focused on five areas of health promotion; as seen in Table 3, these areas coincided with those proposed by [7].

The first study was conducted by [15] and focuses on the ACE program (Activity, Confidence, and Eating). This program promotes healthy eating, physical activity, and dental and mental health and was developed within the framework of the Schools for Health in Europe (SHE) network, and its objective was to improve the implementation of health promotion programs in educational centers. This program promoted the participation of families, students, and teachers through different activities, such as cooking courses, books, dietician support, etc.

The second study focused on the “Classes in Motion” program, evaluated by [27]. The author approached health through physical activity from an integrated approach, without modification of the curricular program. Prior to the implementation of the program, teachers were trained through specific workshops, to provide them with adequate knowledge about health and active teaching methodologies to improve the motivation and safety of their students.

The third study integrated an iron (Fe) deficiency control program, evaluated by [24]. It was a national health promotion program, focused on nutrition and increasing the consumption of Fe supplementation. The importance of this study lies in the fact that the prevalence of Fe deficiency anemia is very high at the global level and, specifically, in Iran affects 35% of the child population, 33% of non-pregnant women, and 40% of pregnant women. Thus, the program focused on a women’s center and was developed in three phases; weekly administration of Fe, monitoring and control, and nutritional information on foods rich in Fe.

The fourth program called the “A Stop Smoking in Schools Trial” (ASSIST) focused on tobacco prevention, as evaluated by [25], and was intended to extend, through the students themselves, information and knowledge on the prevention of tobacco use in all contexts, both school and family. The students received training from health experts and became a trainer and promoter of healthy behaviors and preventive smoking.

The fifth program called “Join the Healthy Boat”, evaluated by [26], focused on the promotion of non-sedentary habits and the responsible use and consumption of ICT. It focused on the importance of training teaching staff in physical activity, healthy diet, and active free time, as well as motivating families to take part in the project. Through the collaboration of families at home, they controlled exercises and activities for the development of healthy habits, especially relevant to preventing sedentary lifestyles in the pandemic period.

The sixth program, called “Active Teen Leaders Avoiding Screen-time” (ATLAS), studied by [27], aimed to improve the frequency of physical activity, reduce the intake of sugary drinks, and reduce the time and consumption of ICT. This program considered that these objectives were achievable by motivating, improving, and reinforcing the individual responsibility of each student in maintaining healthy habits. The intervention was based on the use of multiple resources (physical activity sessions, telephone app, and website for self-monitoring of physical activity).

The last program, called “Hopeful Minds”, evaluated by [28], focused on the mental well-being of students. It proposed the promotion of mental health through social and emotional learning experiences provided by teachers. The study plan was carried out in two phases. In the first, skills such as meditation or managing a journal for self-reflection were taught; in the second, exercise and improvement of these practices.

### 3.4. Effectiveness and Main Difficulties in the Development of Programs That Promote Health

The analysis of the results of the programs has confirmed that not all the programs were effective and not all of them achieved their expected results, as seen in Table 4.

In the study [15], focusing on the primary education stage, the main results showed that teachers considered that schools have a fundamental role in health promotion, they saw work on this issue as incompatible to due to their overload of daily work. For this reason, they suggested that the program should consider the study plan of the center so that it could be implemented without creating more workload for the teaching staff. In addition, due to the lack of training in health, teaching staff considered it very important to have professional support to promote health in an appropriate way. In addition, they stated that one of the most important points was to create and reinforce the bond between the school and family so that students acquire healthy habits.

The study [23] focused on primary school students between the ages of eight and nine. Even though volunteer teachers participated in the study, a fact that guaranteed greater commitment and motivation towards the program, the results were not as expected. Some positive changes were observed in motor skills that led to an improvement in coordination and spatial orientation skills, without other notable results regarding the acquisition of habits.

The results of the study carried out with women in secondary education [24] showed that the three main aspects of the program were not carried out efficiently, since the consumption of the pill due to Fe deficiency (food supplement) was very low and, among the consequences, was a lack of knowledge about health and, specifically, on nutrition, due to the fact that the training sessions were very scarce. Therefore, the program did not achieve its expected objectives.

The research [25], conducted in secondary education, highlighted the importance of peer support in relational activities and social interaction, with support partners benefiting the most. It must be kept in mind that the peer conversations did not penetrate as expected from the students. However, the program still achieved benefits for students, with improvements in self-esteem, communication skills, and group social cohesion. 

The third and last study carried out in primary education [26], showed improvements in development and motivation towards physical activity or sports practice, however, this did not reduce the time dedicated to ICT consumption. In general terms, the program showed some changes, but it was not effective due to the scant participation and collaboration of families in controlling inappropriate habits.

The latest study [27], conducted in secondary education, showed the satisfaction of users with the activities of the program and the place where it was carried out. However, the program was very routine, causing a lack of motivation and interest in the proposed content. The motivation and support of the teachers were key to achieving the proposed objectives. The students became aware of the importance of daily exercise and sedentary behaviors were reduced, the consumption of sugary drinks was reduced, and their diet improved by introducing healthy food. From the empowerment of autonomy in decision-making, it was intended that the students become aware of their role in improving their health.

The study [28], in the stages of primary and secondary education on the effects on the emotional and mental health of students, showed that in the primary stage it was able to reduce anxiety and improve negative emotions, and an improvement in autonomy was evidenced in the management and control of emotions. However, in secondary education no improvements were observed in anxiety levels, although a resilient behavior of students with self-care habits and improved self-confidence was noted.

## 4. Discussion

The main purpose of this systematic review was to determine the effects of programs that promote health in educational centers. This review provides evidence that not all educational programs work, and, in many cases, the expected results are not achieved. However, those that work show positive effects regarding the development of skills, competencies, habits, well-being, etc. The articles reviewed were published between 2016 and 2021, and reflected the importance of educational centers in promoting healthy habits and lifestyles, considering the most effective context for their implementation. The number of studies carried out on programs that promote health has been scarce in recent years. That is why we believe that the development of training programs for the promotion of healthy habits should be promoted; in this sense, we believe that students should be taught to assume a proactive attitude towards the care and maintenance of healthy lifestyles. This is a commitment that requires the involvement of all social agents if we are to consolidate healthy and perpetual lifestyles. Even so, of the studies reviewed, it was found that: (a) the study samples varied and focused mainly on students, with studies focused on teachers and their training in health being especially scarce; (b) there is no clear and concise evaluation method to study health promotion in educational centers; and (c) the results obtained in the studies show variability in the effectiveness of the programs.

In general, not all programs work or generate behaviors that are compatible with the development of healthy habits in students and the rest of the educational community. The results of the systematic review carried out evidenced this fact. Only three of the seven programs obtained expected results, generating positive effects in students towards the development of healthy habits. From the programs presented above, results have been extracted that agree with the study carried out by [17], programs for the promotion of healthy habits have been found to take account of the school’s programming so that they can be carried out effectively, without overloading teachers. The planning and inclusion of health education in the school curriculum give it the continuous and integrated character that any knowledge requires to be acquired and integrated. Programs should tend to move away from isolated interventions in the form of ad hoc campaigns whose effect is insignificant (e.g., oral hygiene day, world sports day…).

Teachers demand the presence of experts to support the implementation of the programs, considering that they do not have sufficient qualifications for this task, and emphasize the importance of families being integrated into the school to achieve real change [15].

The participation of the entire educational community is essential for the success of these programs. As seen in the study [23], motivation of the teaching staff is not enough to achieve beneficial changes with the programs. Similarly, it was observed in the study [26], whose program did not work due to the low participation of families from home. Low family participation conditions the results [29]. When there is involvement and commitment on the part of families in the development of the programs, achievement of the results and goals of the program is favored [30,31].

Programs must be properly structured, focused on health promotion objectives, ensuring sufficient, adequate, and adapted training for students [24]. Specific or isolated interventions not adequately integrated into the training plan and educational programming of the centers do not guarantee the development of habits or the consolidation of self-care behaviors compatible with the health of the students [16]. It is essential that necessary time is dedicated to health promotion, to generate stable changes [18].

The findings of this study have highlighted the importance of having qualified and motivated teachers towards the promotion of health in schools, as well as having the spaces, resources, and materials for its promotion and consolidation [27]. Another study [30] corroborates the value of motivation and interest of the students towards the promotional health programs. The development of this type of program favored peer training, as well as the development of creative, critical, interpersonal thinking, and self-awareness skills that are fundamental for life [25]. Students learn in this way and by putting these skills into practice, to face health-related problems responsibly [31]. Peer learning has been established as a basic strategy for the promotion and consolidation of healthy habits and behaviors [25].

In the study [28], the relevance of training in the success of Health Education Programs was evidenced. Their experience properly developed and achieved optimal changes in the students, greater resilience, improved self-care, and decreased anxiety and negative thoughts were evidenced. Another study [29] valued the importance of the adequate development of health promotion programs, this type of action must be presented in the curricular framework of teaching and their actions must obey a strategic plan of actions focused on the improvement of the health of the students and the educational community. Specific or incomplete actions limiting the phase of action and change that must be carried out by the students and end up being actions without effect.

## 5. Conclusions

The main conclusions reached with this study are:

- Education and health promotion programs in schools must link families with the educational center.
- Improving the training of teachers in health matters is a requirement.
- Health promotion is a social commitment that requires the participation of all its members.
- Health education is not an exclusive commitment of schools, it must involve families and health professionals.
- Health education must be a fundamental objective in the annual programming of schools.
- Programs have to be well structured to work.
- Peer training is beneficial and makes programs work.
- The teacher must be a fundamental support point in the success of health promotion, they must lead the change by encouraging and motivating students towards the adoption of healthy habits.
- An improvement in the qualification and training of teachers in the field of health is required.
- Health promotion programs must be, above all, programs for the training of the entire educational community.

## Figures and Tables

**Table 1:** Estimated inclusion and exclusion criteria.

Inclusion	Exclusion
Studies in English Documents written in the last 10 years Documents that focus on the stage of Early Childhood, Primary and Secondary Education Research articles Studies on health promotion programs Articles that have open access	Articles older than 10 years Articles in languages other than English Articles not focused on health programs Articles that do not base their results on the evaluation of the effectiveness of the programs Articles of reflection

**Table 2:** Primary outcomes of the reviewed articles.

Cite	Purpose	Country	Year	Design	Sample	Assessment Instrument	Type of Program	Primary Outcomes
[15]	Identify the factors that affect the acceptability of health promotion programs in the everyday school environment	Ireland	2016	Qualitative	31 Professors	Semi-structured interviews	Not preventive	Educational centers play a fundamental role in promoting children’s health. But, some aspects of health promotion programs are incompatible with daily school work.
[23]	Evaluate the effectiveness of an integrated health promotion program in the Lower Austrian primary schools based on the Health Promoting Schools framework (HPS) to increase the number of emotional and social experiences, physical activity and wellbeing at school	Austria	2016	Quantitative	432 students participating	- Motor coordination tests - PAQ-C questionnaire. - Questionnaire on social and emotional experiences - KIDSCREEN-52 Questionnaire - Subtests of German Motoric Test - Coordination test for children (KiKo) - Test D2	Not preventive	The intervention does not have a relevant effect on the expected results at the individual level.
[24]	Evaluate the implementation of an Fe supplementation program in secondary schools of the West Azerbaijan province in northwestern Iran; and evaluate the usefulness of the Crosswise Model (CM) to evaluate the health implementation program	Iran	2019	Mixed (quantitative and qualitative)	2180 students (1740 questionnaires and 440 interviews)	- Cross-sectional model questionnaire (CM). - Direct questionnaire (DQ)	Not preventive	The poor quality of program implementation and incomplete and irregular intake of Fe supplements by of high school students made the program ineffective in reducing both Fe deficiency and anemia due to Fe deficiency in this group
[25]	Evaluate the fidelity and acceptability of the study, putting it into practice in a different country and the context for which it was created	Scotland	2019	Mixed (quantitative and qualitative)	2130 students, 41 center staff, 31 trainers and 17 program developers)	- Structured observations. - Questionnaire. - Interviews - Focus groups	Preventive	It is feasible and acceptable to offer the ASSIST smoking prevention program with high-level fidelity beyond the context in which it was developed originally
[26]	Evaluate the effectiveness of the environment-based health promotion program “Join the Healthy Boat” on sedentary time in elementary school children	Germany	2020	Quantitative	231 students (133 from the experimental group and the rest from the control group)	- Multi-sensor device - Questionnaire for parents	Not preventive	The program (“Join the Healthy Boat”) failed to reduce sedentary time within 12 months; this was especially evident on weekends
[27]	Identify whether comments obtained from a representative group of ATLAS participants on their perceptions of the program and its effects reflected the self-determination theory (SDT) basis in the what the program was based on	Australia	2018	Qualitative	42 students	- Interviews in focus groups	Not preventive	There were no significant intervention effects on activity, although changes were seen in time behind screens, muscular endurance, and training skills
[28]	Evaluate a unique program that incorporates resilience, coping, problem-solving and confidence building	United States of America	2019	Mixed (qualitative and quantitative)	88 students (63 from Primary Education and the rest from Secondary)	- Interviews in focus groups - Spence Children’s Questionnaire - How I Feel Scale - Gad-7 Questionnaire - Scale of Difficulties in Emotional Regulation-Short form - Adolescent Resilience Questionnaire	Preventive	The general findings suggest that this theoretically framed hope-based program was able to significantly improve levels of anxiety and emotional regulation in elementary school students and improve adaptive coping strategies and resilience in post-primary students

**Table 3:** Areas of health promotion.

Cite.	Healthy Nutrition	Physical Activity	Emotional Wellbeing	Consumption	Ambient
[15]	Yes	Yes	Yes	-	-
[23]	-	Yes	-	-	Yes
[24]	Yes	-	-	-	-
[25]	-	-	-	Yes	-
[26]	Yes	Yes	-	Yes	-
[27]	Yes	Yes	-	Yes	-
[28]	-	-	Yes	-	-

**Table 4:** Effectiveness of health promotion programs.

Cite	Effectiveness	Main Difficulties
[15]	No	Excess work, little qualification of the teaching staff, and little family participation
[23]	No	Inappropriate intervention
[24]	No	Inadequate planning and implementation of the program
[25]	Yes	-
[26]	No	Lack of family participation
[27]	Yes	-
[28]	Yes	-

## Data Availability

Information and queries on the data used can be obtained from this article.
